# The International Journal of Health Policy and Management (IJHPM) in 2024: Progress and Innovation

**DOI:** 10.34172/ijhpm.9087

**Published:** 2025-04-23

**Authors:** Mina Moradzadeh, Vahid Yazdi-Feyzabadi, Sahar Najafizadeh, AliAkbar Haghdoost

**Affiliations:** ^1^Rehabilitation Research Center, Department of Rehabilitation Basic Sciences, School of Rehabilitation Sciences, Iran University of Medical Sciences, Tehran, Iran.; ^2^Health Services Management Research Center, Institute for Futures Studies in Health, Kerman University of Medical Sciences, Kerman, Iran.; ^3^HIV/STI Surveillance Research Center, and WHO Collaborating Center for HIV Surveillance, Institute for Futures Studies in Health, Kerman University of Medical Sciences, Kerman, Iran.

## Introduction

 Towards the end of 2024, it is an opportune time to reflect on the progress of the *International Journal of Health Policy and Management* (IJHPM) over the past year and outline our plans for 2025.

## Reflections on the IJHPM in 2024

 Throughout 2024, IJHPM remained committed to publishing high-quality research in health policy and management, maintaining rigorous double-blind peer-review, and upholding the highest standards of scientific integrity. Over the year, a total of 142 articles were published, comprising 136 articles in the regular volume and six articles in a special issue titled “Pakistan’s Progress on Universal Health Coverage.” Table provides an overview of the published articles by type.

**Table:** The Frequency and Type of Articles Published in the 2024 Issue of the *International Journal of Health Policy and Management*

**Type of Article**	**Regular Issue**	**Special Issue**	**Total**
Editorial	5	1	6
Review Article	13	0	13
Short Communication	4	0	4
Original Article	50	5	55
Commentary	47	0	47
Correspondence	8	0	8
Viewpoint	2	0	2
Letter to Editor	7	0	7
**Total**	**136**	**6**	**142**

## Contributions From Authors in 2024

 In 2024, the IJHPM received contributions from 610^[1]^ authors across 49 countries, including first-time contributors from Ivory Coast, Libya, Palestine, Senegal, and Yemen. The highest number of published articles came from the United Kingdom (n = 122), followed by Canada (n = 86) and Australia (n = 63). A detailed breakdown of author contributions by country is provided in Table S1 (Supplementary file 1).

## Contributions From Reviewers in 2024

 A total of 459 reviewers from 62 countries participated in IJHPM’s peer-review process in 2024. Their invaluable contributions are deeply appreciated. The majority of reviews came from the United States (n = 83), followed by China (n = 58) and Australia (n = 45). A comprehensive list of reviewers by country is available in Table S2 (Supplementary file 1).

## Role of the Editorial Board in 2024

 The Editorial Board played a crucial role in IJHPM’s achievements in 2024, actively participating in the review process, shaping journal policies, and authoring insightful editorials.

 As indicated in Table, six editorials were published in 2024. One of these, contributed by guest editors of the special issue, provided an evidence-based framework for essential health services package in primary healthcare, emphasizing health system gaps, stakeholder engagement, capacity building, and international collaboration to improve priority-setting frameworks like DCP3 according to Universal Health Coverage.^1^ The remaining five editorials, authored by IJHPM Editorial Board members, addressed critical topics in global health and policy (Dr. Eivind Engebretsen, Dr. Martin McKee, Dr. Jo Rycroft-Malone, Dr. Naoki Ikegami, and Dr. Ronald Labonté).

 Eivind Engebretsen and Mona Baker, in “The Rhetoric of Decolonizing Global Health Fails to Address the Reality of Settler Colonialism: Gaza as a Case in Point”^2^ called for a reassessment of global health institutions within the neoliberal framework and the colonial biases inherent in knowledge production.

 Martin McKee and his colleagues, in “Placing Trust at the Heart of Health Policy and Systems,”^3^ underscored the importance of trust in health systems, advocating for its measurement, maintenance, and restoration.

 Jo Rycroft-Malone and her colleagues, in “Research Coproduction: An Underused Pathway to Impact,”^4^ highlighted the necessity of collaborative research involving both researchers and non-researchers to enhance impact, discussing the principles of co-production across different system levels.

 Naoki Ikegami, in “The Economic Rationale for Healthcare Reform,”^5^ examined the economic justification for healthcare access, the efficiency of labor division in healthcare, and inconsistencies in cost-effectiveness assessments for pharmaceutical pricing and prescribing.

 Ronald Labonté, in “Can a Well-Being Economy Save Us?,”^6^ explored economic reforms in response to global challenges such as climate change, addressing critical questions: (1) Are some governments advancing quickly and sufficiently towards a “well-being economy”? (2) What policy actions are necessary for governments? (3) How can they counter the powerful interests resisting changes threatening their privileges?

## IJHPM’s Citation Metrics

**Figure 1:**
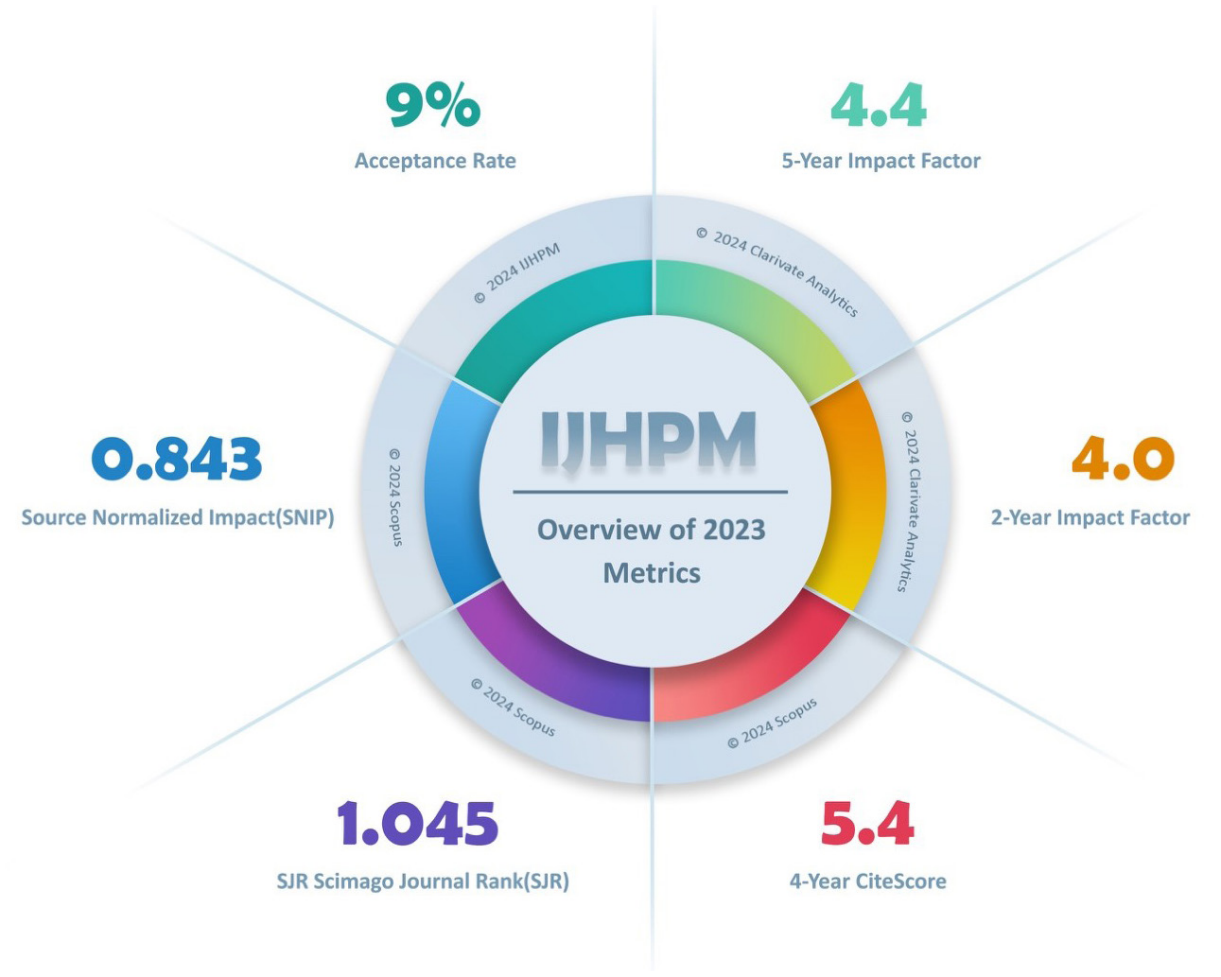
Overview of International Journal of Health Policy and Management Metrics.

**Figure 2:**
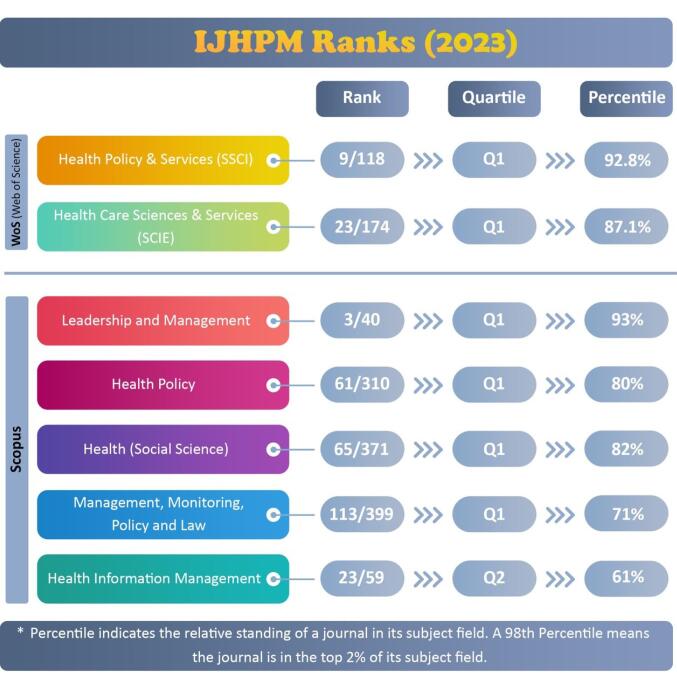
International Journal of Health Policy and Management Ranks Based on Clarivate Web of Science and Scopus.

 Beyond publishing high-quality research, IJHPM has achieved impressive citation metrics and global rankings. According to Clarivate Analytics’ Journal Citation Report 2023 (JCR 2023), released in June 2024, our impact factor remains strong (IF 2023: 4.0), positioning IJHPM in the top quartile (Q1) of Health Policy and Healthcare journals. Additionally, our CiteScore increased from 4.7 to 5.4, reflecting a rise in article citations. Figures 1 and 2 provide a detailed overview of IJHPM’s ranking in Clarivate Web of Science and Scopus. Based on our close monitoring of citation trends, we anticipate a growth in IJHPM’s metrics in the forthcoming Clarivate Web of Science and Scopus reports.

 Overall, these metrics reflect the high-quality of research published in IJHPM and our commitment to advancing the health policy and management field. By mentioning that the journal is in the top quartile (Q1) of Health Policy and Management journals in both Clarivate Web of Science and Scopus, it emphasizes the journal’s prestige and influence in the field.

 Despite these achievements, challenges remain. One such instance involved an author requesting anonymous publication. As a member of Committee on Publication Ethics (COPE)^[2]^, IJHPM consulted the COPE Council before proceeding with the publication.^7^

## IJHPM Plans for 2025

 Looking ahead, IJHPM will continue to uphold its commitment to publishing high-quality research while broadening its global reach. Since its inception in June 2013 through December 2024, IJHPM has published contributions from 6889 authors across 115 countries. The United Nations^[3]^ currently recognizes 195 countries^[4]^, therefore 80 nations have yet to be represented in the IJHPM’s publications. To expand our international scope and foster cross-cultural knowledge exchange, we actively encourage researchers from these underrepresented countries (Table S3, Supplementary file 1) to contribute to IJHPM.

 A key initiative for 2025 will be developing guidelines on using artificial intelligence (AI) in research. These guidelines will adhere to the standards set by the International Committee of Medical Journal Editors (ICMJE) and COPE. Additionally, we will implement internal guidelines to familiarize our editors with AI-related ethical considerations and assist them in evaluating AI-related issues during the pre-screening and peer-review processes.

## Conclusion

 IJHPM remains committed to advancing health policy and management research throughout 2025. With continued collaboration from our authors, reviewers, and editorial board, we aim to strengthen our impact, uphold the highest academic standards, and foster a more inclusive global research community.

## Ethical issues

 Not applicable.

## Conflicts of interest

 Authors declare that they have no conflicts of interest. However, It should be noted that all of the authors are in-house editors at the *International Journal of Health Policy and Management.*

## Endnotes


^[1]^ Repeated authors are counted, so one author may contribute more than once in 2024.
^[2]^ https://publicationethics.org/members/international-journal-health-policy-and-management.
^[3]^ https://www.un.org/en/about-us/member-states.
^[4]^ Including two non-member observer states: the Vatican City and the State of Palestine.

## Supplementary files


Supplementary file 1 contains Tables S1-S3.

## References

[1] Alwan A, Jamison DT, Siddiqi S, Vassall A (2024). Pakistan’s progress on universal health coverage: lessons learned in priority setting and challenges ahead in reinforcing primary healthcare. Int J Health Policy Manag.

[2] Engebretsen E, Baker M (2024). The rhetoric of decolonizing global health fails to address the reality of settler colonialism: Gaza as a case in point. Int J Health Policy Manag.

[3] McKee M, van Schalkwyk MC, Greenley R, Permanand G (2024). Placing trust at the heart of health policy and systems. Int J Health Policy Manag.

[4] Rycroft-Malone J, Graham ID, Kothari A, McCutcheon C (2024). Research coproduction: an underused pathway to impact. Int J Health Policy Manag.

[5] Ikegami N (2024). The economic rationale for healthcare reform. Int J Health Policy Manag.

[6] Labonté R (2024). Can a well-being economy save us?. Int J Health Policy Manag.

[7] Anonymous Anonymous (2024). Are burned babies and mass graves a global health crisis? What does decolonization got to do with it? Comment on “The rhetoric of decolonizing global health fails to address the reality of settler colonialism: Gaza as a case in point. ” Int J Health Policy Manag.

