# Supplementary material for: The International Journal of Health Policy and Management (IJHPM) in 2024: Progress and Innovation
Source: Int J Health Policy Manag. 2025 Apr 23;14:9087. doi: 10.34172/ijhpm.9087 (PMC12257198; doi:10.34172/ijhpm.9087)
Supplement: Supplementary file 1 — contains Tables S1-S3. [file ijhpm-14-9087-s001.pdf]

Article title: The International Journal of Health Policy and Management (IJHPM) in 2024: Progress and Innovation

Journal name: International Journal of Health Policy and Management (IJHPM)

Authors' information: Mina Moradzadeh[1*], Vahid Yazdi-Feyzabadi[2], Sahar Najafizadeh[2], AliAkbar Haghdoost[3*]

[1]Rehabilitation Research Center, Department of Rehabilitation Basic Sciences, School of Rehabilitation Sciences, Iran University of Medical Sciences, Tehran, Iran.

[2]Health Services Management Research Center, Institute for Futures Studies in Health, Kerman University of Medical Sciences, Kerman, Iran.

[3]HIV/STI Surveillance Research Center, and WHO Collaborating Center for HIV Surveillance, Institute for Futures Studies in Health, Kerman University of Medical Sciences, Kerman, Iran.

***Correspondence to:** Mina Moradzadeh, Email: Moradzadeh.mina@iums.ac.ir & AliAkbar Haghdoost, Email: ahaghdoost@kmu.ac.ir

Citation: Moradzadeh M, Yazdi-Feyzabadi V, Najafizadeh S, Haghdoost AA. The International Journal of Health Policy and Management (IJHPM) in 2024: progress and innovation. *Int J Health Policy Manag*. 2025;14:9087. doi:10.34172/ ijhpm.9087

Supplementary file 1

Table S1: Frequency of IJHPM Authors Per Country in 2024

	Country Name	Number of Authors
1	United Kingdom	115
2	Canada	86
3	Australia	63
4	China	58
5	Netherlands	50
6	Pakistan	38
7	USA	30
8	Iran	17
9	Republic of Korea (South Korea)	15
10	Italy	9
11	South Africa	9
12	Thailand	8
13	Belgium	7
14	Finland	7
15	Sweden	7

16	Israel	6
17	Malaysia	6
18	Austria	5
19	Uganda	5
20	Japan	4
21	Malawi	4
22	Cameroon	3
23	Congo	3
24	Hungary	3
25	India	3
26	Ireland	3
27	Kazakhstan	3
28	Norway	3
29	Palestine	3
30	Portugal	3
31	Switzerland	3
32	Egypt	2
33	France	2
34	Mexico	2
35	New Zealand	2
36	Nigeria	2
37	Denmark	1
38	Ethiopia	1
39	Germany	1
40	Ivory Coast	1
41	Kenya	1
42	Libya	1
43	Morocco	1
44	Nepal	1
45	Philippines	1
46	Senegal	1
47	Singapore	1
48	Spain	1
49	Yemen	1
	**Total**	**602**

Table S2: Frequency of IJHPM Reviewers Per Country in 2024

	Country Name	Number of Reviewers
1	United States	83
2	China	58
3	Australia	45
4	United Kingdom	42
5	Canada	34
6	Iran	24
7	Netherlands	14
8	India	12
9	Republic of Korea	11
10	Spain	9
11	Italy	8
12	Nigeria	7
13	South Africa	7
14	Germany	6
15	Ireland	6
16	Japan	5
17	Norway	5
18	Switzerland	5
19	Brazil	4
20	Ghana	4
21	Pakistan	4
22	Poland	4
23	Austria	3
24	Egypt	3
25	France	3
26	Indonesia	3
27	Saudi Arabia	3
28	Sweden	3
29	Denmark	2
30	Ethiopia	2
31	Finland	2
32	Kenya	2
33	Mexico	2
34	Philippines	2
35	Singapore	2
36	Thailand	2
37	Turkey	2
38	Uganda	2
39	Bangladesh	1

40	Barbados	1
41	Belgium	1
42	Bosnia and Herzegowina	1
43	Botswana	1
44	Bulgaria	1
45	Colombia	1
46	Croatia	1
47	Czech Republic	1
48	Ecuador	1
49	Fiji	1
50	Greece	1
51	Hungary	1
52	Iceland	1
53	Israel	1
54	Kuwait	1
55	Luxembourg	1
56	Malawi	1
57	Malaysia	1
58	Palestine	1
59	Portugal	1
60	Romania	1
61	Tanzania	1
62	Zimbabwe	1
	**Total**	**459**

Table S3: List of Countries with No Publications in the International Journal of Health Policy and Management (IJHPM)

	**Country Name**
1	Albania
2	Andorra
3	Angola
4	Antigua and Barbuda
5	Armenia
6	Azerbaijan
7	Bahamas
8	Belarus
9	Belize
10	Bolivia (Plurinational State of)
11	Bosnia And Herzegovina
12	Burundi

13	Cape Verde (Cabo Verde)
14	Central African Republic
15	Chad
16	Comoros
17	Democratic People's Republic of Korea (North Korea)
18	Djibouti
19	Dominica
20	Dominican Republic
21	Ecuador
22	Equatorial Guinea
23	Eritrea
24	Eswatini
25	Fiji
26	Gabon
27	Gambia (Republic of The)
28	Georgia
29	Grenada
30	Guinea Bissau
31	Guyana
32	Honduras
33	Iceland
34	Iraq
35	Jamaica
36	Kiribati
37	Kyrgyzstan
38	Lesotho
39	Liberia
40	Liechtenstein
41	Lithuania
42	Madagascar
43	Marshall Islands
44	Mauritania
45	Mauritius
46	Micronesia (Federated States of)
47	Monaco
48	Montenegro
49	Namibia
50	Nauru
51	Nicaragua

52	Niger
53	Palau
54	Panama
55	Paraguay
56	Republic of Moldova
57	Romania
58	Saint Kitts And Nevis
59	Saint Lucia
60	Saint Vincent And The Grenadines
61	San Marino
62	Sao Tome And Principe
63	Serbia
64	Seychelles
65	Slovenia
66	Solomon Islands
67	Sri Lanka
68	Suriname
69	Syrian Arab Republic (Syria)
70	Tajikistan
71	Timor-Leste
72	Togo
73	Tonga
74	Turkmenistan
75	Tuvalu
76	Ukraine
77	Uruguay
78	Uzbekistan
79	Vatican City
80	Venezuela, Bolivarian Republic of
